# Comparison of Efficacy and Safety of Different Guided Technologies Combined With Ultrathin Bronchoscopic Biopsy for Peripheral Pulmonary Lesions

**DOI:** 10.1111/crj.70012

**Published:** 2024-09-27

**Authors:** Zhihan Zhang, Junbao Zhang, Xi Chen, Junfeng Yan, Cao Zeng, Ping Xu

**Affiliations:** ^1^ Department of Pulmonary and Critical Care Medicine Peking University Shenzhen Hospital Shenzhen Guangdong China; ^2^ Peking University Health Science Center Beijing China; ^3^ Shantou University Medical College Shantou Guangdong China; ^4^ University of Michigan Ann Arbor Michigan USA

**Keywords:** diagnose, guidance, navigation, peripheral pulmonary lesions, safety, ultrathin bronchoscopy

## Abstract

**Introduction:**

Various bronchoscopic guidance techniques have emerged to improve the diagnostic yield of peripheral pulmonary lesions (PPLs), especially when combined with ultra‐thin bronchoscopy. However, uncertainties exists in the convenience, accuracy rate, and complications of these techniques. We compared the feasibility, accuracy rate, and complication rates of transbronchial biopsy of PPLs sampled by the standard thin‐layer CT navigation combined with ultrathin bronchoscopy (CTNUTB), the Lungpro virtual navigation combined with ultrathin bronchoscopy (VNUTB), and electromagnetic navigation combined with ultrathin bronchoscopy (ENUTB).

**Methods:**

Retrospectively identified were 256 patients sampled with transbronchial biopsy of PPLs. Eligible patients referred for CTNUTB, VNUTB, and ENUTB from January 2017 to December 2021 were included. We comprehensively compared the accuracy rate, feasibility, and complication rates for each method.

**Results:**

There was no significant difference in the accuracy rate of CTNUTB, VNUTB, and ENUTB (*p* = 0.293). The operation time via Lungpro navigation was the shortest (14.4 min, *p* < 0.001). The planning time via CT planning was the shortest (7.36 min, *p* < 0.001). There was no difference in the incidence of complications such as hemorrhage, pneumonia, and pneumothorax (*p* = 0.123). Besides, ENUTB costs more than $2000, while CTNUTB and VNUTB cost only about $130–230.

**Conclusion:**

CTNUTB is still the main bronchoscopy method we recommended, which has low cost, simple operation, and safety no less than the others. In contrast, ENUTB provides a higher accuracy rate for small diameter nodules (less than 2 cm), which has a high use value and is worth promoting in the future.

## Graphical abstract

We aim at comparing different guided technologies combined with bronchoscopic biopsy for peripheral pulmonary lesions. Standard thin‐layer CT navigation combined with ultrathin bronchoscopy is still the main bronchoscopy method we recommended, which has the advantages of low cost, simple operation, and safety no less than others. Besides, bronchoscope under electromagnetic navigation provides a high diagnostic yield for small diameter nodules.

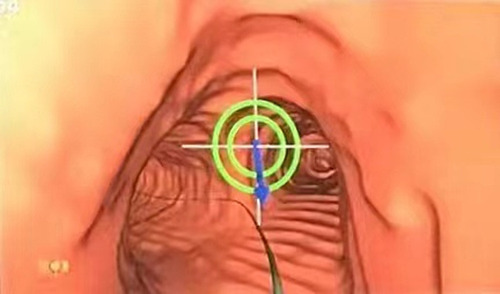

**Abbreviations**
CTNUTB: standard thin‐layer CT navigation combined with ultrathin bronchoscopy
ENUTB: electromagnetic navigation combined with ultrathin bronchoscopy
PPLs: peripheral pulmonary lesions
UTB: ultrathin bronchoscopy
VNB: the Lungpro virtual navigation bronchoscopy
VNUTB: the Lungpro virtual navigation combined with ultrathin bronchoscopy

## 1 Introduction

Peripheral pulmonary lesions (PPLs) are generally defined as nodules (< 3 cm) or masses (> 3 cm) around the outer third of the lung. With the emergence of new imaging methods and the increasing incidence of pulmonary nodules [1], the examination demand and detection rate of pulmonary lesions have increased [2, 3]. Bronchoscopy is the most commonly accepted diagnostic method for PPLs, considering the incidence and severity of complications of the percutaneous approach were significantly higher [4, 5]. However, due to the lack of direct visualization and a relatively low malignancy rate [6], sampling guided by regular bronchoscopy, especially for small pulmonary nodules that <2 cm, has a low probability of successful sampling. The sensitivity of bronchoscopy in diagnosing peripheral lung cancer < 20 mm was only 34% [7]. With the improvement of bronchoscopy and its auxiliary technologies, it is gradually recommended to use technologies to make safe and effective tissue diagnosis of PPLs, for example, the standard thin‐layer CT navigation combined with ultrathin bronchoscopy (CTNUTB), the Lungpro virtual navigation combined with ultrathin bronchoscopy (VNUTB), electromagnetic navigation combined with ultrathin bronchoscopy (ENUTB), and robotic‐assisted bronchoscopy navigation [8, 9]. But the benefits of these are still controversial.

With the development of CT technology and reconstruction technology on this basis, CT plays a more important role in the diagnosis of pulmonary nodules and bronchoscopic guidance. Thin‐layer CT can show more details of PPLs than conventional CT [10], which can guide bronchoscopy to reach the corresponding bronchial lumen of the lesion. However, due to the inability to obtain three‐dimensional images and the change of the position of the trachea structure causing by breathing and posture changes, this method may lead to repeated CT scanning [11] and the operator entering the wrong operation path [12], which can be improved by using virtual navigation technology [13].

Virtual bronchoscopy (VB) is a 3D display technology [14], and the Lungpro virtual navigation bronchoscopy (VNB) is a method of creating the 3D images of bronchial pathways that match CT and using them as navigation [15]. The biggest disadvantage of VNB is that it is not real time, which is completely dependent on CT data before bronchoscopy. A mucus plug that blocks the airway may prevent creating a direct path to the target lesion. Enlargement of pleural effusion may also change the location of the lesion.

ENUTB is a technology that is based on electromagnetic positioning technology, combining with high‐resolution CT imaging and computer virtual bronchoscope technology [16, 17]. Its tilt, rotation, and other movements in the electromagnetic field can be acquired by the positioning system and transmitted to the computer [18]. A meta‐analysis found that electromagnetic navigation system is superior to Lungpro virtual navigation in the diagnosis of lung lesions, which may be due to the real‐time positioning function of ENUTB [19]. However, ENUTB's operation steps of localization are relatively complicated, and the inspection cost is relatively expensive. Besides, respiratory movement and the placement of the bronchoscopy may also cause the displacement of the lesions [18].

The differences between the three navigation methods mentioned above in terms of diagnosis rate and complication rate are still unclear. In order to study the application value and difference of CTNUTB, VNUTB, and ENUTB, 263 samples were included to explore the feasibility, accuracy rate, and safety of the three techniques.

## 2 Materials and Methods

### 2.1 Research Object

This is a single‐center retrospective study. We selected 263 eligible patients from January 2017 to December 2021 who received CTNUTB, VNUTB, and ENUTB in the Department of Respiratory Medicine of Peking University Shenzhen Hospital after exclusion of contraindications, including 156 males and 96 females, aged 17–87 years, with an average age of 54.59 (15.9) years. The study protocol was approved by the Ethics Review Committee, and all study participants obtained informed consent for study.

### 2.2 Data Collection

**FIGURE 1:**
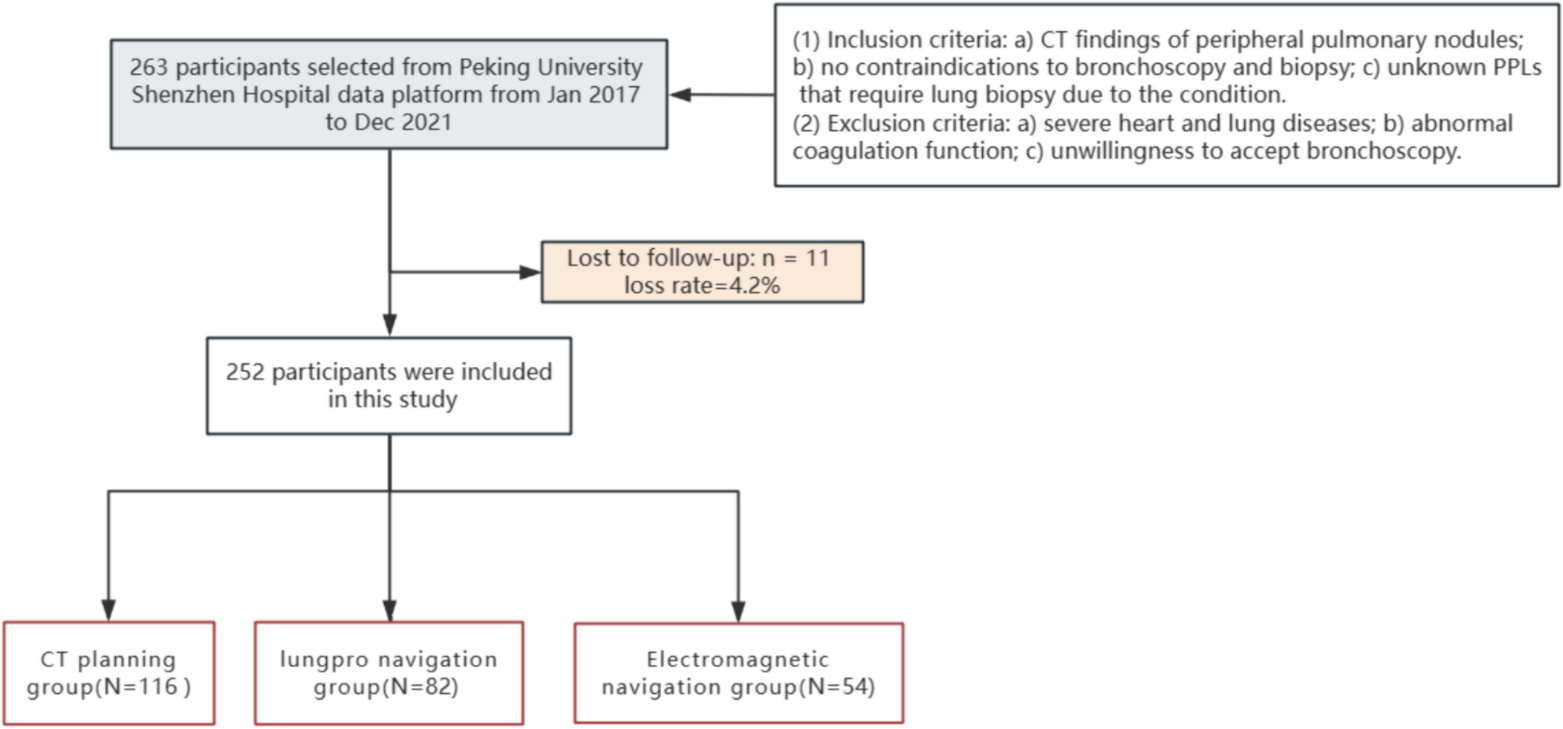
Flow diagram of the screening and enrollment of study participants.

This study retrospectively investigated the patients who had received bronchoscopy recorded in the electronic medical record and included the patients who had received standard CTNUTB, VNUTB, and ENUTB biopsy and showed PPLs on CT. Inclusion criteria for patients: (1) Inclusion criteria: (a) CT findings of PPLs around the outer third of the lung, (b) no contraindications to bronchoscopy and biopsy, (c) unknown PPLs that require lung biopsy due to the condition; (2) exclusion criteria: (a) severe heart and lung diseases, (b) abnormal coagulation function, and (c) unwillingness to accept bronchoscopy. Included in the study analysis according to the time sequence of patients’ visits. According to different methods of route planning, patients were divided into three groups: CT planning group, Lungpro navigation group, and electromagnetic navigation group. According to the pathological diagnosis results of the biopsy samples, the patients' lesions were determined to be tumor and non‐tumor nodules, and the patients who were determined to be non‐tumor by biopsy were followed up for 2 years. The resulting cohort included 252 participants for the final analysis (Figure 1).

### 2.3 Research Procedures

All bronchoscopic procedures were based on the fact that there were no absolute contraindications in blood routine examination, blood coagulation test, electrocardiogram and chest CT before operation, and basic anesthesia (generally, fentanyl citrate 12.5 μg, Midazolam 2.5 mg, the patient was conscious, and the patient was able to breathe autonomously) was used. The dosage varies according to the physical condition of different patients, as assessed by the anesthesiologist. At the same time, 4% lidocaine injection (25 mL) was used for local anesthesia. All these bronchoscopic procedures are performed through the mouth, without an endotracheal tube or fluoroscopy. During the operation, the healthy side was first entered, and then the affected side was entered to observe each branch of the bronchus and clear the airway secretions. Finally, the lesion site was explored, biopsy forceps (instead of needles, fluid lavage, brush) were placed, and the clamp was repeated 4 to 6 times (the number of biopsies was determined according to the size of the biopsy tissue, the amount of blood lost after biopsy, and the patient's tolerance), without fluoroscopy. Device information for the three technologies is presented in Table 1.

**TABLE 1:** Device information for the three technologies.

Category	UTB model	Guided technology model
CTNUTB	Flexible bronchoscopy (BF‐XP 290, 3.1 mm outside diameter, Olympus, Tokyo, Japan)	Thin‐layer CT (layer thickness smaller than or equal to 0.625 mm)
VNUTB	Flexible bronchoscopy (BF‐XP 290, 3.1 mm outside diameter, Olympus, Tokyo, Japan)	The LungPoint system
ENUTB	Flexible bronchoscopy (BF‐XP 290, 3.1 mm outside diameter, Olympus, Tokyo, Japan)	The Langkai (Suzhou, China) system

#### 2.3.1 CTNUTB

According to 64‐slice multi‐slice spiral CT scan (layer thickness is 0.625 mm), the doctor carefully examined and determined the bronchial path leading to the lesion. During the operation, the bronchoscope was pushed towards the interested subsegment, and the sampling equipment (via the bronchial biopsy forceps) was pushed to the location of lesions.

#### 2.3.2 VNUTB

**FIGURE 2:**
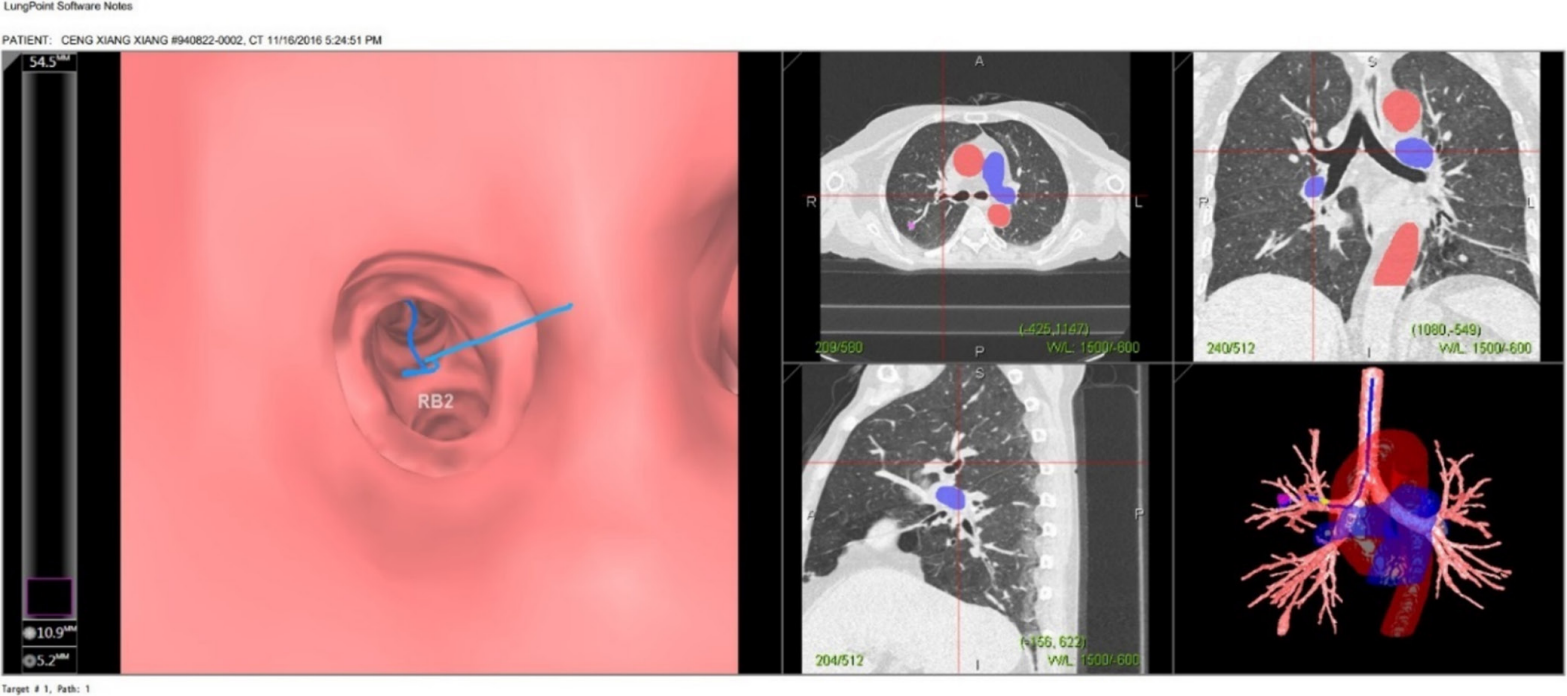
VNUTB virtual image shows airway and main blood vessels with diameter ≥ 3 mm (lower right).

VNUTB uses 64‐slice multi‐slice spiral CT scan (layer thickness is 0.625 mm) to generate a three‐dimensional virtual image of the airway, aligning and superimposing the virtual image on the actual bronchoscopic view at the same time. The commercial system of Lungpro virtual navigation we use is LungPoint System (Figure 2). After obtaining CT, the system displays the airways larger than 3 cm in diameter and main blood vessels by calculating the central axis of each airway. In the process of bronchoscopy, real‐time bronchoscopy and VB images are synchronized (although VB images are not real time). This synchronization requires assistants to slowly advance and adjust VB images.

#### 2.3.3 ENUTB

**FIGURE 3:**
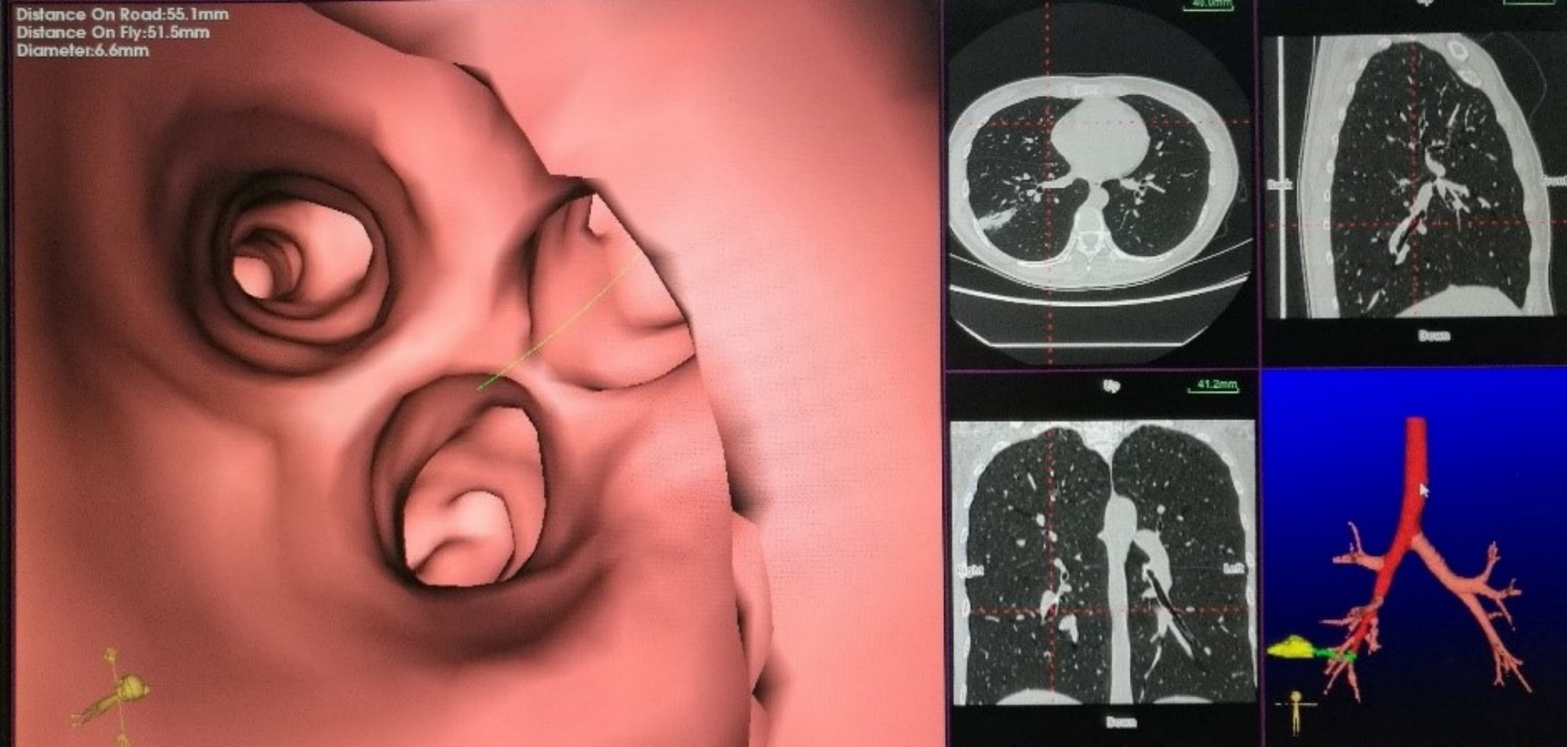
ENUTB draws a virtual bronchial tree (lower right).

ENUTB technology also requires preoperative CT (64‐slice multi‐slice spiral CT scan, layer thickness is 0.625 mm) to create a virtual bronchial tree. During operation, the ENUTB equipment generates electromagnetic field around the patient's chest, and a small electromagnetic sensor is used as the position locator in the field. The catheter with electronic sensor at the tip can initially be pushed in all parts of the airway to synchronize and match the virtual airway with the actual anatomical structure. The system automatically registers the electromagnetic position information. After the registration, the bronchial tree is green. You check the matching degree, if not satisfied, can re‐register. The system can perform automatic registration. After successful registration, the target positioning sensor is oriented towards the lesion, then we can locate and sample the target lesion. The commercial electromagnetic navigation system we use is Langkai (Suzhou, China) (Figure 3), which has obtained CFDA product registration certificate and obtained EU ce certification.

#### 2.3.4 Diagnosis

Histological and cytological samples were explained in detail by specialized histopathologists and cytopathologists, respectively. If the pathological result is a tumor, the pathological diagnosis is confirmed. If the pathological result is a non‐tumor, it needs to be followed up for 2 years to reevaluate whether it is consistent with the clinical and imaging findings.

Planning time is defined as the time when CT data are imported into the planning system for route planning before navigation. For the CT planning group, planning time is defined as the time for physicians to plan bronchoscopy routes. Operation time is defined as the time from the beginning to the completion of bronchoscopy, and the measurement unit is minute. The total time is the navigation‐planning time plus the operation time, and the measurement unit is minute. The complications of the three bronchoscopic techniques are defined as whether there is hemorrhage, pneumonia, and pneumothorax during the operation or within 48 h after the operation. The criteria for hemorrhage are local injection of ice cold saline, adrenaline, or balloon closure is required to stop bleeding. Those who need vascular intervention or surgical intervention to stop bleeding or need to be admitted to the ICU. If there is no bleeding or the bleeding can be stopped by negative pressure suction, the bleeding without other hemostatic measures will not be included in the bleeding. Measurement unit: 0 means no complication, 1 means complication.

#### 2.3.5 Study Objectives

1. The main objective of the study: The main objective of the study is to investigate some important indicators of diagnostic tests of PPLs by CTNUTB, VNUTB, and ENUTB biopsy. According to the final clinical diagnosis results after follow‐up, we can divide the bronchoscopy biopsy results into three parts: yes, no, and missing, and remove the missing part, which is a very small part. We calculated important indicators of diagnostic tests in each navigation mode, including area under the curve, accuracy, sensitivity, specificity, and so forth.
2. Secondary objective of the study: To explore the navigation‐planning time, total operation time, and the complication rate of CTNUTB, VNUTB, and ENUTB biopsy PPL. Among them, for PLs of different sizes, we also explore the accuracy rate and intra‐group differences of each method; for PLs of the same size, we explore the difference between groups in accuracy rate of different operation methods.

## 3 Statistical Analysis

The software SPSS 23.0 and R version 4.2.0 were used for statistical analysis. The data are expressed in terms of average with standard deviation or number of cases with percentage. *t*‐test or ANOVA or Wilcoxon rank sum test are used for statistical tests of continuous variables. Fisher exact test is used for statistical test of classified variables. Primary end point: The accuracy rate is equal to the number of cases in which the clinical diagnosis was consistent with the bronchoscopic biopsy results divided by the total number. The chi square test of independent samples was used for the three groups of statistical tests. Fisher exact test was used to compare the accuracy of three groups with lesions of different sizes. Secondary end point: The operation time, planned time, and total time were calculated in accordance with the normal distribution, and the test method was *t*‐test or analysis of variance. Fisher exact test was used to test the complications. Results were considered significant when the *p* value was less than or equal to 0.05.

## 4 Result

During the study period, 252 subjects (116 using CT planning, 82 using Lungpro navigation, and 54 using electromagnetic navigation) were examined by bronchoscopy. Baseline characteristics of the three groups are similar (Table 2). The average age (standard deviation, SD) of the study population (156 males and 96 females) was 54.59 (15.9) years old. The most common lesion site was the right upper lobe (91/252, 36.1%), followed by the left lower lobe (45/252, 17.9%). The lobular distribution of lesions was similar among the three groups (Table 2).

**TABLE 2:** Demographic profile of the patients and characteristics of lesions.

Parameter	CT planning group	Lungpro navigation group	Electromagnetic navigation group	*p*.overall	*p*_1_	*p*_2_	*p*_3_
Number	*N* = 116	*N* = 82	*N* = 54				
Age	55.1 (15.0)	54.3 (17.0)	53.9 (16.5)	0.898	0.948	0.902	0.988
Gender				0.103	0.21	0.587	0.197
Female	47 (40.5%)	24 (29.3%)	25 (46.3%)				
Male	69 (59.5%)	58 (70.7%)	29 (53.7%)				
Lesion size	3.32 (0.68)	3.06 (1.01)	2.63 (0.92)	< 0.001	0.092	< 0.001	0.012
Lesion location					0.455	0.06	0.455
LLL	29 (25.0%)	19 (23.2%)	7 (13.0%)				
LUL	15 (12.9%)	16 (19.5%)	11 (20.4%)				
RLL	14 (12.1%)	15 (18.3%)	16 (29.6%)				
RML	9 (7.76%)	6 (7.32%)	4 (7.41%)				
RUL	49 (42.2%)	26 (31.7%)	16 (29.6%)				
Specific findings					0.001	0.053	0.484
Adenocarcinoma	23 (19.8%)	22 (26.8%)	18 (33.3%)				
Squamous cell carcinoma	17 (14.7%)	14 (17.1%)	11 (20.4%)				
NSCLC	26 (22.4%)	5 (6.10%)	4 (7.41%)				
SCLC	0 (0.00%)	6 (7.32%)	1 (1.85%)				
Other malignancy	5 (4.31%)	0 (0.00%)	1 (1.85%)				
Nonmalignancy	45 (38.8%)	35 (42.7%)	19 (35.2%)				
Specific findings‐team				0.674	0.778	0.778	0.778
Benign team	45 (38.8%)	35 (42.7%)	19 (35.2%)				
Malignant team	71 (61.2%)	47 (57.3%)	35 (64.8%)				

*Note: t*‐test or ANOVA or Wilcoxon rank sum test are used for statistical tests of continuous variables. FISHER exact test is used for statistical test of classified variables. The function of “Specific findings‐team” is classified as the endoscopic diagnosis.

Abbreviations: LLL: left lower lobe; LUL: left upper lobe; NSCLC: non‐small cell lung carcinoma; *p*.overall: *p* value obtained by comparison of three groups; *p*_1_: *p* value obtained by comparing CT planning group with Lungpro navigation group; *p*_2_: *p* value obtained by comparing CT planning group with electromagnetic navigation group; *p*_3_: *p* value obtained by comparing Lungpro navigation group with electromagnetic navigation group; RLL: right lower lobe; RML: right middle lobe; RUL: right upper lobe; SCLC: small cell lung carcinoma.

**FIGURE 4:**
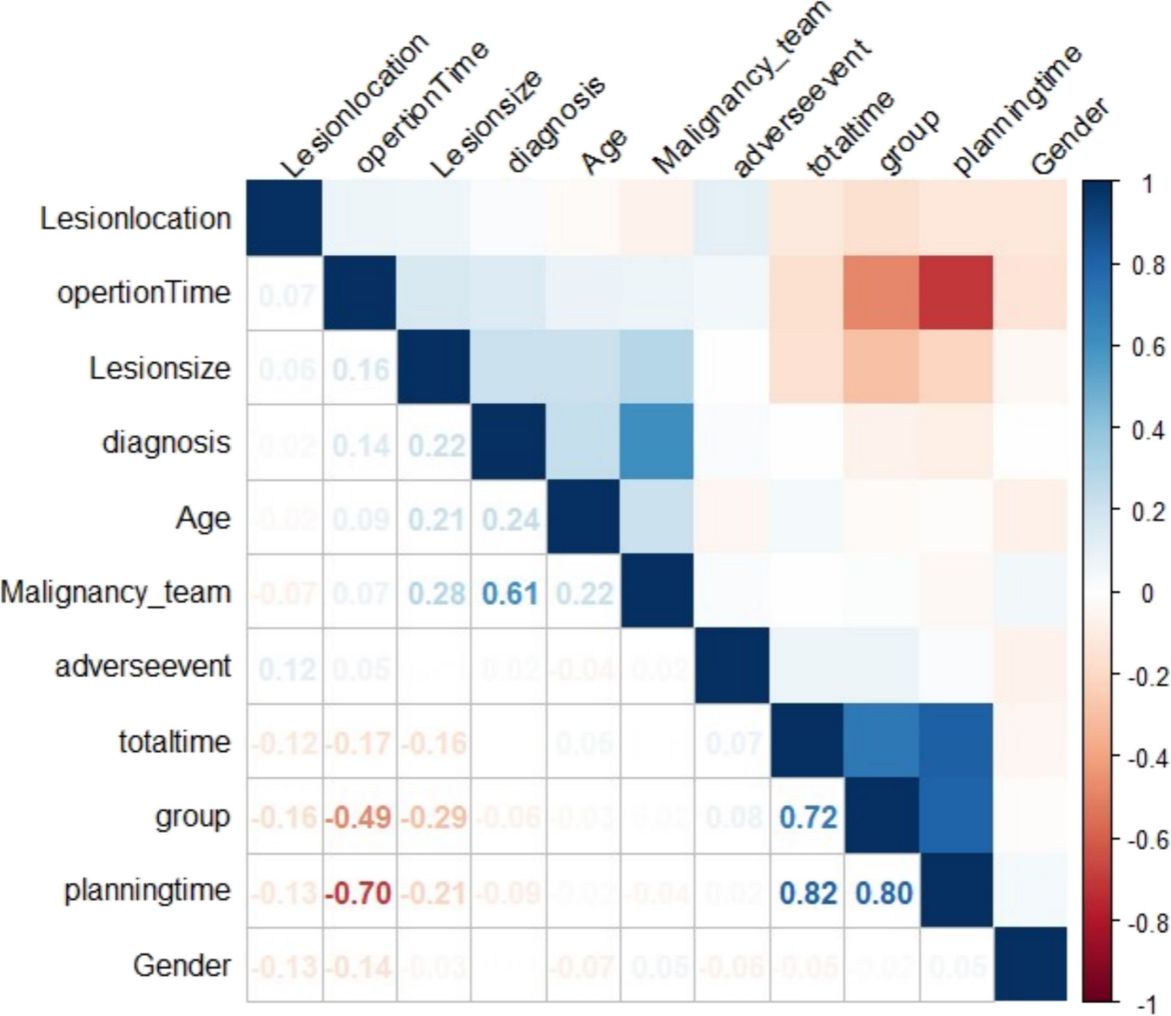
Correlation coefficient diagram of variables.

From the correlation coefficient graph, it can be found that grouping is highly correlated with navigation time and total time, with correlation coefficients of 0.80 and 0.72, respectively (Figure 4).

Primary end point: The overall diagnostic yield was 75.4% according to the histological diagnosis. Among the three groups, the final clinical diagnosis was malignant in 84.5%, 75.6%, and 79.6% of cases, respectively (*p* = 0.293, independent sample chi square test) (Table 3).

**TABLE 3:** The diagnosis of the three groups.

Parameter	CT planning group	Lungpro navigation group	Electromagnetic navigation group	*p*.overall	*p*_1_	*p*_2_	*p*_3_
Diagnosis				0.293	0.504	0.735	0.735
Benign	18 (15.5%)	20 (24.4%)	11 (20.4%)				
Malignant	98 (84.5%)	62 (75.6%)	43 (79.6%)				

*Note:* The function of “diagnosis” is classified as the final clinical diagnosis. The chi square test of independent samples was used for the three groups of statistical tests.

Abbreviations: *p*.overall: *p* value obtained by comparison of three groups; *p*_1_: *p* value obtained by comparing CT planning group with Lungpro navigation group; *p*_2_: *p* value obtained by comparing CT planning group with electromagnetic navigation group; *p*_3_: *p* value obtained by comparing Lungpro navigation group with electromagnetic navigation group.

Finally, it was found that the accuracy rate of the three groups was 76.7%, 81.7%, and 85.2% (*p* = 0.9039, independent sample chi square test) (Table 4). The AUC values of the three groups were 0.862, 0.879, and 0.907, respectively.

**TABLE 4:** Important indicators of diagnostic tests of the three groups.

	CT planning group	Lungpro navigation group	Electromagnetic navigation group	*p*.overall	*p*_1_	*p*_2_	*p*_3_
Accuracy rate	0.767	0.817	0.852	0.9039	0.8556	0.7612	0.9755
Area under the curve	0.862	0.879	0.907				
Sensitivity	0.724	0.758	0.814				

*Note:* Accuracy rate is equal to (true positive + true negative)/sample population, the same as “the number of cases in which the clinical diagnosis was consistent with the bronchoscopic biopsy results divided by the total number.” Sensitivity is equal to the diagnostic yield for malignant lesions. The chi square test of independent samples was used for the three groups of statistical tests.

Abbreviations: *p*.overall: *p* value obtained by comparison of three groups; *p*_1_: *p* value obtained by comparing CT planning group with Lungpro navigation group; *p*_2_: *p* value obtained by comparing CT planning group with electromagnetic navigation group; *p*_3_: *p* value obtained by comparing Lungpro navigation group with electromagnetic navigation group.

Secondary end point: In terms of operation time, the Lungpro navigation group was the shortest (*p* < 0.001). In terms of planning time, CT planning group was shortest (*p* < 0.001). CT planning group was shortest in total time. There was no significant difference in the incidence of hemorrhage, pneumonia, and pneumothorax (*p* = 0.123) (Table 5). We roughed up the cost of different directions. Staff costs are calculated according to doctor rank and salary. The cost of the machine is calculated according to the time cost, the cost of the equipment itself, and the consumables for each inspection. In general, ENUTB costs more than $2000, and CTNUTB costs only about $130, which includes CT examination costs ($100) and bronchoscopy costs ($30). Besides, VNUTB costs $100 more than CTNUTB (Lungpro navigation costs), whose total cost is about $230.

**TABLE 5:** Other outcome parameters of the three groups.

Parameter	CT planning group	Lungpro navigation group	Electromagnetic navigation group	*p*.overall	*p*_1_	*p*_2_	*p*_3_
Operation time	23.0 (3.68)	14.4 (2.93)	18.4 (2.84)	< 0.001	< 0.001	< 0.001	< 0.001
Planning time	7.36 (1.57)	24.4 (3.50)	22.4 (3.07)	< 0.001	< 0.001	< 0.001	< 0.001
Total time	30.4 (4.02)	38.8 (4.27)	40.9 (3.79)	< 0.001	< 0.001	< 0.001	0.011
Adverse event				0.123	0.333	0.274	0.312
No	105 (90.5%)	77 (93.9%)	48 (88.9%)				
Hemorrhage	8 (6.90%)	2 (2.44%)	1 (1.85%)				
Pneumonia	1 (0.86%)	0 (0.00%)	3 (5.56%)				
Pneumothorax	2 (1.72%)	3 (3.66%)	2 (3.70%)				
Cost	$130	$230	More than $2000				

*Note:* The operation time, planned time, and total time were calculated in accordance with the normal distribution, and the test method was *t*‐test or analysis of variance. Fisher exact test was used to test the complications.

Abbreviations: *p*.overall: *p* value obtained by comparison of three groups; *p*_1_: *p* value obtained by comparing CT planning group with Lungpro navigation group; *p*_2_: *p* value obtained by comparing CT planning group with electromagnetic navigation group; *p*_3_: *p* value obtained by comparing Lungpro navigation group with electromagnetic navigation group.

The electromagnetic navigation group is relatively superior in diagnostic yield (*p* = 0.293) (Table 6). Under the same size, CTNUTB, VNUTB, and ENUTB biopsy have no significant difference in the diagnostic yield. Under the same operation method, the diagnostic yield have no statistical difference, either. However, ENUTB is more accurate for nodules smaller than 2 cm (CT planning group versus electromagnetic navigation group with a nodule size of 0–2 cm *p* is 0.027), although there was no statistical difference between VNUTB and ENUTB.

**TABLE 6:** The accuracy of different guided technologies of different nodule size.

	Diagnostic yield (less than 2 cm)	Diagnostic yield (2–3 cm)	Diagnostic yield (more than 3)	*p*_5_
CT planning group	0.5 (4/8)	0.73 (44/60)	0.85 (41/48)	0.682
Lungpro navigation group	0.71 (15/21)	0.80 (21/26)	0.89 (31/35)	0.870
Electromagnetic navigation group	0.86 (19/22)	0.87 (20/23)	0.78 (7/9)	1
*p*_4_	0.625	0.908	1	

*Note:* The accuracy of the three groups with different size of lesions was compared by Fisher test. For the comparison between CT planning group and electromagnetic navigation group with a nodule size of 1–2 cm, the Fisher test *p* is 0.027.

Abbreviations: *p*_4_: for the same size, the accuracy *p* value among different guided technologies; *p*_5_: for the same guided technology (the same group), the accuracy *p* value of different sizes.

## 5 Discussion

As far as we know, this is the first retrospective study to report the effectiveness of the joint comparison of CTNUTB, VNUTB, and ENUTB. Our study finally proved that the diagnostic yield for malignant lesions of PPLs with CTNUTB was as high as 72.4%. This is the same as the diagnostic yield (60%–78.3%) [20, 21, 22, 23, 24, 25] of PPLs sampled by various bronchoscopes reported by many researches since the report on the diagnosis of PPLs by the bronchoscope with an outer diameter of 3.5 mm, which was published in 1985 [26]. A study compared the diagnostic yields of PPLs by CT guided and electromagnetic navigation guided transthoracic biopsy, which were 86.0% and 66.0%, respectively [27]. We think the ultrathin bronchoscope can go deeper into the distal bronchus and improve the diagnostic accuracy of peripheral lung cancer compared with the traditional bronchoscope, which is consistent with another study [28]. Besides, navigation system also proves its potential. These auxiliary technologies improve the selection of bronchial routes to lesions [29] and the location of small lesions. A meta‐analysis also showed that the combined diagnostic yield of electromagnetic navigation, virtual navigation, and other guided bronchoscopy was as high as 70% [30].

Our study found that there was no difference in the proportion of nodules detected at different locations (left lung and right lung, upper lobe and middle lobe and lower lobe) in PPLs, which was consistent with other studies [31, 32]. The benign and malignant nodules diagnosed by endoscope were significantly correlated with the actual benign and malignant nodules. The overall malignant ratio was 80.6%. When talking about the size of nodules, a retrospective study [33] and a meta study [34] suggested that it was an important factor affecting the diagnostic yield. However, it has also been reported that the size of the lesion is not a significant independent variable of the diagnostic yield of bronchoscopy [31, 32]. The latter is consistent with our research results. In terms of planning time and overall time, the planning time of CTNUTB was only 7.36 min, and the total time was only 30.4 min, which was significantly shorter than that of VNUTB and ENUTB (*p* < 0.001). In addition, our study also found that there was no statistical difference in the incidence of complications among the three groups. Besides, ENUTB costs more than $2000, while CTNUTB costs only about $130 and VNUTB costs $230.

There are many researches comparing VNB with non‐VNB. A prospective multicenter study found that the accuracy of VNB group in diagnosing PPLs was higher than that of non‐VNB group (80% vs. 67%; *p* = 0.032), and the operation time was shortened by about 2 min [35]. Another study also showed the advantage of VNB in operation time (20.6 ± 12.8 min vs. 28.6 ± 14.3 min, *p* = 0.023) [36]. However, a prospective randomized multicenter trial showed that there was no significant difference between VNB and non‐VNB in the diagnosis of PPL of less than 30 mm (67% vs. 60%; *p* = 0.173) [37]. In another study, there is no difference between the VNUTB and unguided arms in overall diagnostic yield (47% vs. 40%, *p* = 0.354) [38]. Our study further proves that VNUTB is not superior in the diagnosis yield and total time of PPLs compared with CTNUTB and ENUTB.

Bronchoscopy under CT planning is a recommended method for biopsy of peripheral lung diseases. Considering practical conditions, economic analysis [39], and our research results, CT planning has the advantages of high accessibility, high feasibility, low cost‐beneficial, short operation time, and safety no less than that of navigation bronchoscopy. ENUTB is another recommended method for diagnosing peripheral lung diseases. Electromagnetic navigation is becoming more and more popular in North America and Europe [40]. The third edition of the evidence‐based clinical practice guidelines of the American College of Chest Physicians pointed out that electromagnetic navigation is recommended for peripheral lesions that are difficult to reach only through conventional bronchoscopy [4]. Although the diagnostic yield of electromagnetic navigation varies greatly, including 59% to 94% [41], this may be related to the difference in operator experience, selection bias, and the unclear use standard of it. Our research suggests that ENUTB (rather than CTNUTB and VNUTB) can improve the accuracy of small nodules (0–2 cm), which can reduce the number of surgeries for small nodules in the future as the technology is promoted and the cost is reduced.

The current research has several limitations. The first limitation is that our bronchoscopy is performed by experienced experts. As we all know, any bronchoscopy procedure involves a personal learning curve [42], so the bronchoscopy personnel with less experience may not be able to fully use CT to imagine three‐dimensional images and choose the correct path. In order to obtain satisfactory results in bronchoscopy, more operators with different ages may be required to operate and observe the proficiency of the examiner. The second limitation is that the sample size is insufficient. The data with less than 10 samples (such as the number of samples in the electromagnetic navigation group) is difficult to represent the whole population. The reason is that the ENUTB has high price of navigation and positioning wires, and the medical insurance do not cover it, which leads to the selection bias of patients (especially patients with large nodules clinically) and doctors in our hospital.

In conclusion, CTNUTB is still the main bronchoscopy method we recommended, which has the advantages of low cost, simple operation, and safety no less than VNUTB and ENUTB. CTNUTB is something that physicians should master and should be promoted. In contrast, the biopsy method using ultrathin bronchoscope under electromagnetic navigation provides a high diagnostic yield for small diameter nodules (less than 2 cm) in non‐emergency situations, which has a high use value and is worth promoting in the future. Further studies may be needed to clarify the most appropriate bronchoscopy method for different individuals in the subgroup in more detail.

## Author Contributions

Zhihan Zhang was responsible for experimental design and writing, while Junbao Zhang and Xi Chen were responsible for collecting and organizing data. Junfeng Yang is responsible for analyzing the data. Cao Zeng was responsible for revising the article. Ping Xu is responsible for revising articles and submissions.

## Ethics Statement

The experimental protocol was established, according to the ethical guidelines of the Helsinki Declaration and was approved by the Human Ethics Committee of Peking University Shenzhen Hospital. Written informed consent was obtained from guardian participants. IRB approval number: 20180207, approval date: 2018.02.07.

## Conflicts of Interest

The authors declare no conflicts of interest.

## Data Availability

We selected 263 eligible patients from January 2017 to December 2021 who received CTNUTB, VNUTB, and ENUTB in the Department of Respiratory Medicine of Peking University Shenzhen Hospital after exclusion of contraindications, including 156 males and 96 females, aged 17–87 years, with an average age of 54.59 (15.9) years. The datasets used and analyzed during the current study are available from the corresponding author on reasonable request.
